# The usefulness of biotyping in the determination of selected pathogenicity determinants in *Streptococcus mutans*

**DOI:** 10.1186/1471-2180-14-194

**Published:** 2014-08-05

**Authors:** Wirginia Krzyściak, Katarzyna K Pluskwa, Jakub Piątkowski, Paweł Krzyściak, Anna Jurczak, Dorota Kościelniak, Anna Skalniak

**Affiliations:** 1 Department of Medical Diagnostics, Jagiellonian University, Medical College, Pharmacy Faculty, Medyczna 9, 30-688 Krakow, Poland; 2 Genetics Laboratory, Department of Endocrinology, Jagiellonian University, Medical College, Kopernika 17, 31-501 Krakow, Poland; 3 Department of Mycology, Microbiology, Jagiellonian University, Medical College, Czysta 18, 31-121 Krakow, Poland; 4 Department of Pediatric Dentistry, Institute of Dentistry, Jagiellonian University, Medical College, Montelupich 4, 31-155 Krakow, Poland

**Keywords:** Prephenate dehydrogenase (PDH), Biotyping, Childhood caries (CC), *Streptococcus mutans*

## Abstract

**Background:**

*Streptococcus mutans* is known to be a primary etiological factor of dental caries, a widespread and growing disease in Polish children. Recognition of novel features determining the pathogenicity of this pathogen may contribute to understanding the mechanisms of bacterial infections.

The goal of the study was to determine the activity of prephenate dehydrogenase (PHD) and to illuminate the role of the enzyme in *S. mutans* pathogenicity. The strains were biotyped based on STREPTOtest 24 biochemical identification tests and the usefulness of biotyping in the determination of *S. mutans* pathogenicity determinants was examined.

**Results:**

Out of ninety strains isolated from children with deciduous teeth fifty three were classified as *S. mutans* species. PDH activity was higher (21.69 U/mg on average) in the experimental group compared to the control group (5.74 U/mg on average) (*P* <0.001). Moreover, it was demonstrated that biotype I, established basing on the biochemical characterization of the strain, was predominant (58.5%) in oral cavity streptococcosis. Its dominance was determined by higher PDH activity compared to biotypes II and III (*P* = 0.0019).

**Conclusions:**

The usefulness of biotyping in the determination of *Streptococcus mutans* pathogenicity determinants was demonstrated. The obtained results allow for better differentiation of *S. mutans* species and thus may contribute to recognition of pathogenic bacteria transmission mechanisms and facilitate treatment.

## Background

### Etiology of caries

According to the latest reports, caries should be perceived as a multi-factor disease; four stages are needed for its development. The basic stage includes psychosocial, behavioral and genetic factors, the second level includes interactions between bacteria and the host, and also the influence of sugar in the diet, the third level includes biochemical changes - fermentation of carbohydrates to acids, leading to the fourth level, i.e. pH decrease and enamel demineralization [1].

It has been demonstrated in Polish studies that falling asleep with a bottle, snacking between meals, eating sweets and the sweetening of food in a bottle are factors initiating development of the disease, significantly affecting the increase in the amount of *Streptococcus mutans* bacteria, which is believed to be an important etiological factor for caries development [2].

The fact that for over 20 years the dental health of children in Poland has not improved, seems to be worrying, and the ratio of children affected by caries of deciduous teeth has remained at about 54% [3]. The incidence rate of caries is expressed by the prevalence of caries and its intensity is illustrated by dmf (decayed, missing, filled – for primary teeth, and an average DMF index for permanent teeth). DMF indexes are different depending on the age of children, place of residence, and socio-economic conditions of parents, and may also depend on the sex of the child. Despite public education in the prevention of caries, the problem of dental caries still remains a current topic.

The pathogenic potential of *S. mutans* cariogenic bacteria [4,5] is connected with the ability to metabolize a wide range of sugars, to form biofilm and to create an acidic environment [6]. One of the key intermediate products in *S. mutans* metabolism is pyruvate, and intracellular pH is maintained *via* this compound connected to amino acid synthesis. Researches demonstrated that biosynthesis of branched chain amino acids (valines, leucines, isoleucines) is one of the components of adaptation to an acidic environment [7]. Also, a correlation was noted between tolerance towards low pH and biofilm formation [8]. Since *S. mutans* is accepted to be an etiological caries factor, attempts to find a substance inhibiting cell proliferation have been undertaken. According to Ku et al., targeting the pathways of aromatic amino acid biosynthesis, especially prephenate dehydrogenase (PDH, EC 1.3.1.12), the enzyme catalyzing transition of prephenate into 4-hydroxyphenylpyruvate, may inhibit proliferation of *S. mutants *[9].

The pathway of aromatic amino acid biosynthesis is important in the metabolism of bacteria, fungi and plants and the PDH enzyme as a key component of the pathway may be essential for the pathogens’ survival in the host. Auxotrophic towards amino acids *Mycobacterium tuberculosis* strains lost the ability to proliferate and survive inside macrophages. Bearing in mind the above reports, the PDH enzyme may be examined in the context of being a potential target for therapeutic agents [10]. The structure and enzymatic activity of PDH is being intensively analyzed worldwide. Inter-species differences in the protein structure and features characteristic for enzymes are being identified. Hydrolytic activity was also observed in pathogens like *Haemophilus inluenzae *[11], *Neisseria gonorrhoeae *[12] or *Escherichia coli *[13].

### Typing of *Streptococcus* strains

In the literature, one can find many methods for typing S*treptococci* strains. These are methods based on colony morphotype, resistance to various chemicals, enzymograms, as well as more advanced techniques of typing, namely PCR fingerprinting.

The easiest way to type S*treptococci* strains is morphotyping. This is based on the evaluation of the appearance of colonies on blood agar. Color, edge, surface, the result of Gram staining, and the type of hemolysis are assessed [14].

One of the earliest methods introduced for the phenotyping of S*treptococci* is serotyping, which is based on agglutination. The most effective serotyping has been developed for β-hemolytic *Streptococci*: *S. pyogenes* and *S. agalactiae *[15]; however, no accurate differentiation of strains for epidemiological purposes is possible [16].

In auksanogram-based typing, intraspecific differences in the ability to assimilate different organic compounds are assessed. Depending on the compounds used, different modifications of this method are available. Typing of *S. mutans* strains based on auksanograms and enzymograms using tests like STREPTOtest (Lachema, Pliva) or API Strep (bioMerieux) is quite popular. For STREPTOtest 24, the discrimination ability for the correct identification of strains has been estimated by the producer at 62.2%, which is acceptable from a clinical point of view. In the phenotyping of *Streptococcus viridans* with the use of the API 32 assay from bioMerieux, discrimination ability was estimated at 79% [17].

In a study conducted by Brtkova et al. [18], biochemical assays from Lachema, Pliva and bioMerieux were compared with genetic methods for *Enterococcus* species isolated from animals. Identification carried out with the use of the En-coccus assay (Pliva, Lachema) resulted in the classification of 47 species, out of a total of 97, as *Enterococcus faecium*; 20 randomly selected samples identified as *E. faecium* using the En-coccus assay, were identified again using the API20Strep assay (bioMerieux), and for 8 species, identification was not confirmed. Of the 47 pre-selected strains, 24 were classified to the *E. faecium* species with the use of genetic methods, thus confirming the results obtained with the application of biochemical methods.

The differences between biochemical assays were explained by differences in the number of specific substrates: the En-coccus assay contains only 8 sugars, while the API20 Strep contains 20, which allows for better discrimination [18]. Teles et al. compared commercial API Rapid 32 ID Strep and Vitek 2 in the context of the identification of *Streptococcus viridans*. 79% of the strains were correctly identified based on the API assay, while 19% were correctly classified for the phylogenetic group; the Vitek assay was able to correctly characterize 55% of identified species; for 21% of the species, the phylogenetic group was correctly determined [17].

Another method of typing is biotyping, based on selected enzymatic reactions of commercial assays. This kind of differentiation has been carried out for different species of *Streptococcus*. Barnham et al. identified *S. zooepidemicus* using a commercial API 20 Strep assay, and next, based on the results of reactions, sensitivity to penicillin, erythromycin and tetracycline, they distinguished five biotypes of the species [19]. Aarestrup and Jensen conducted biotyping of *Streptococcus dysagalactiae* basing on two reactions with the use of the commercial Rapid 32 Strep assay for this purpose: production of acid from tegatose and sorbitol, so that four biotypes of the species were observed. In parallel, based on the assessment of specific DNA fragments, ribotypes were defined, and the obtained bio- and ribotypes were consistent [20].

In the studies of Polish researchers, strains of B Streptococci were differentiated using four hydrolysis reactions of sodium hippurate, lactose, salicin and trehalose. Based on this method, eight biotypes, which do not correlate with known serotypes, were distinguished. According to the authors, the investigated biochemical typing may have a similar usefulness as serotyping in the differentiation of group B streptococci [21]. Biotyping has also found an application in distinguishing Streptococci from the group *Streptococcus mutans* (*S. mutans* and *S. sobrinus*) isolated from plaque. Yoo et al. conducted biotyping based on the fermentation profile of mannitol, sorbitol, raffinose, melibiose and arginine decomposition. They classified strains of *S. mutans* to existing biotypes with the following frequencies: 65.26% - biotype I; 10.53% - biotype V; 2.11% - biotype IV; 1.05% - biotype II. In their study, two strains of other biotypes were also observed [22].

Except for the phenotyping methods for *Streptococcus* strains described above, there are techniques based on DNA analysis – i.e. genotyping. The most common methods, with respect to their usage for *Streptococcus sp.* strains, are presented below.

Pulsed field gel electrophoresis (PFGE) is a method of electrophoretic karyotyping. Electrophoretic separation of nuclear DNA occurs in an alternating electric field. This method has been used for the karyotyping of clinical strains of *S. pneumoniae *[23], *S. pyogenes *[24], *S. canis *[25] and *S. mutans *[26]. It appears to be consistent with RAPD typing and auksonograms. It is described as the “gold standard of genetic fingerprints”, but rather time-consuming (due to the difficulties in destroying the cell membrane) [26].

Repetitive extragenic palindromic PCR (rep-PCR) can be successfully used for genotyping *S. mutans*. It is based on a PCR reaction with the use of primers for repetitive sequences, followed by the comparison of the presence or absence of specific bands in agarose gel electrophoresis. Compared to the PFGE method, it is less expensive and less time consuming – thus, more practical for large-scale epidemiological studies [26].

Another method for the assessment of “DNA-fingerprinting” is the analysis of restriction fragment length polymorphism (RFLP). A PCR reaction is performed, and then the amplification product is treated with a restriction enzyme. After electrophoresis, characteristic bands are analyzed. RFLP-PCR is used for genetic studies of *Streptococcus*, e.g. *S. pneumoniae* species [27], *S. bovis*/*S.equinis *[28] or *S. mutans *[29].

The PCR method and its different variants, depending on the purpose for which they are used, are the most common methods for the typing of pathogenic bacteria. Moser et al. compared the genetic methods for epidemiological studies and concluded that the rep-PCR method exhibits sufficient quality at a relatively low cost [26]. Other genetic studies on pathogenic bacteria are, in turn, based on RFLP-PCR [28], or sequencing of the 16S rRNA subunit [17]. There are various methods for the molecular identification and typing of bacteria, and most are based on the PCR reaction, although amplified fragments and even the subsequent course vary depending on the method.

### Aim of the study

This study undertook an attempt to determine the degree of the metabolism of aromatic amino acids by *S. mutans* on the basis of one of the key enzymes of the aromatic amino acid pathway - prephenate dehydrogenase. This enzyme catalyzes the reaction of prephenate transition into 4-hydroxyphenylpyruvate and is not observed in humans [9], and therefore could be a target for newly synthesized compounds of potential antibacterial activity. In the present study, an attempt at strain biotyping based on the STREPTOtest 24 test was undertaken, and the usefulness of biotyping in determination of *Streptococcus mutans* pathogenicity determinants was examined.

Our study aimed to identify bacteria present in caries and to find an enzyme or an enzymatic biotype that could be targeted to hinder bacterial survival and therefore to prevent caries development.

## Methods

### Subject of the study

The study included strains isolated from patients (n = 61; age: 6 ± 1.9 years) with diagnosed early childhood caries (ECC) of deciduous teeth, treated stomatologically during the period from February to April 2012 at the Department of Pediatric Dentistry, Institute of Dentistry, Jagiellonian University, Medical College, Krakow, Poland. The control group was formed of strains isolated from children (n = 29, age: 6 ± 1.5 years) without caries lesions of deciduous teeth, who were kept under the clinic’s control (Table 1).

**Table 1:** Demographic characteristics of the subjects

**Outcome variable**	**Mean ± SD in group**		***P***
	**ECC (n = 61)**	**CF (n = 29)**	
Age (yr)	6 ± 1.9	6 ± 1,5	0.37^a^
Sex, number			0.75^b^
Female number %	31 (51)	12 (41)	
Male number %	30 (49)	17 (59)	
Caries status dmft^c^ score	6.5 ± 2.5	0	

ECC: early childhood caries; CF: caries free.

^a^By the nonparametric Mann–Whitney *U* test for independent samples.

^b^By the Fisher’s exact test.

^c^dmft, decayed, missing, and filled teeth.

The classification of patients for the study was performed by a qualified dentist during a routine dental examination. Dental examinations were based on WHO recommendations. The legal guardians of the participants of the study were fully informed about its aim and course, and agreed on their childrens’ participation. The factors excluding from participation in the study were insufficiently developed chewing activity observed in small children and mentally disabled patients, and the lack of an agreement from legal guardians for their childrens’ participation in the experiment.

### Material collection

The specimens were collected from carious foci in teeth of children with caries, and from dental plaque from children without caries. After classification of the patients to the research, scrapings from carious foci (in the case of children with caries) or from dental plaque (in children without caries) were collected for examination using an open system composed of a sterile cotton swab placed in a test tube. The patients maintained a fasting status, and sampling was performed between 8-10 a.m. after prior oral cavity rinsing with distilled water. The collected material was sealed in sterile test tubes, inhibiting oxygen access, and transported at room temperature to the laboratory within a time period of no longer than 1 hour.

### Culture of collected material on HLR-S medium

The material was inoculated on a selective medium (HLR-S) in the laboratory. The range of inhibition of the growth of bacteria colonizing an oral cavity was verified in a preliminary study on four media, which are described in the literature as selective for this kind of pathogens [30,31]. The results are presented in Table 2.

**Table 2:** Comparison of culture media for enumeration of oral streptococci

	**HLR-S**	**BA**	**SMM**	**TYCSB**	***P***
Control	-	-	-	-	
Mixed strains^g^	++	+++	+	+++	
Dental plaque^h^	+	+++	-	+++	
Carious foci^i^	+	++	+	++	
*S. mutans*^a^	+++	+++	-	+++	0.027^j^
*S. sanguis*^b^	-	+++	+	++	0.031^k^
*S. aureus*^c^	-	+++	++	+++	0.025^l^
*S. pyogenes*^d^	-	++	-	+	0.017^ł^
*H. influeznae*^e^	-	-	-	-	
*S. pneumoniae*^f^	-	+++	-	-	0.006^m^

HLR-S: the modified medium of Ritz; BA: bacitracin agar; TYCSB: trypticase-yeast extract-cysteine sucrose-bacitracin agar.

- no growth of bacteria.

+ 3 × 10^2^ CFU/ml.

++ 2 × 10^3^ CFU/ml.

+++ > 3 × 10^4^ CFU/ml.

^a^reference strain ATCC 700610.

^b, d, e, f^clinical strains.

^c^reference strain ATCC 259232.

^g^Contains: *S. mutans*, *S. sanguis*, *S. aureus*, *S. pyogens*, *S. pneumonia*, *H. influenzae* in phosphate buffered saline (0.5 McFarland each).

^h^Averaged from 20 samples.

^i^Averaged from 20 samples.

^j^By the parametric Student-*t* test for independent samples.

^k^By the parametric Student-*t* test for independent samples.

^l^By the nonparametric Mann–Whitney *U* test for independent samples.

^ł^By the parametric Student-*t* test for independent samples.

^m^ By the parametric Student-*t* test for independent samples.

### Bacterial identification

#### Biotyping of the isolated strains

Enzymatic typing was performed based on the enzymatic profiles obtained from the STREPTOtest 24 test (Lachema, Pliva). The enzymes contained in the test are presented in Table 3.

**Table 3:** Enzymatic assay

**Designation (test code)**	**Studied enzyme**	**Reaction**
NAG	N-acetyl-glucosaminidase	Hydrolisys
LAP	L-leucin-aminopeptidase	Hydrolisys
bMN	β-mannosidase	Hydrolisys
GLR	β-glucuronidase	Hydrolisys
bGL	β-glucosidase	Hydrolisys
bGA	β-galactosidase	Hydrolisys
Aga	α-galactosidase	Hydrolisys
PHS	Phosphatase	Hydrolysis of compound
ESL	Eskulin	Hydrolysis of compound
INU	Inulin	Fermentation of sugar
MAN	Mannitol	Fermentation of compound
SOR	Sorbitol	Fermentation of compound
MLB	Mellibiose	Fermentation of sugar
RIB	Ribose	Fermentation of sugar
LAC	Lactose	Fermentation of sugar
PUL	Pullulan	Fermentation of sugar
ARG	Arginin	Hydrolysis of compound
S06	Growth in 6.5% NaCl	
AMG	α-methylglucosidase	Hydrolisys
TGT	Tagatose	Fermentation of sugar
MLT	Maltose	Fermentation of sugar
RAF	Rafinose	Fermentation of sugar
TRE	Trehalose	Fermentation of sugar
SOE	Sorbose	Fermentation of sugar

The strains were initially cultured on HLR-S medium for 48 hours at a temperature of 36°C in the presence of 10% CO_2_. The bacteria were then inoculated onto blood agar (BA) medium, and incubated for 48 hours at 36°C in the presence of 10% CO_2_. The suspension of pure colonies of a density of 2-2.2 on the McFarland scale was prepared in 3-3.5 ml sterile physiological saline. 100 μl of this bacterial suspension was put to each of the wells of the first row (labeled: NAG, LAP, bMN, GLR, bGL, bGA, Aga, PHS) and to the wells in column H (labeled: ESL i ARG). 1.5 ml of the suspension of a density of 2-2.2 on the McFarland scale was transferred into physiological saline as a suspension carrier for the STREPTOtest 24 (Lachema, Pliva). 100 μl of the prepared suspension was put into each of the rest of the reaction wells (INU, MAN, SOR, MLB, RIB, LAC, PUL, S06, AMG, TGT, MLT, RAF, TRE, SOE). A drop of paraffin oil was added to the wells labeled ARG and S06.

After transferring the prepared solutions, a STREPTOtest 24 test incubation was performed for 24 hours at 37°C. The read-out was made visually based on a comparative color scale attached to the test; the reaction results were interpreted as “positive” or “negative”. The results were presented in a 7-point scale (0-7) depending on reaction intensity. The species were identified based on a code-book provided by the manufacturer (suitable species are ascribed to the codes).

#### ***Genotyping of the isolated strains***

##### 

**DNA preparation** Four colonies from each monoculture in BA medium (10%, 36°C) were suspended in 20 μl water (Nuclease free water; Ambion, Life Technologies, USA; No P/N AM9938). The suspension was heated for 15 minutes in 100°C on a heating block.

The samples were centrifuged for 3 minutes at 6,000 × g [RCF] in 4°C, with a break speed of zero. Fifteen μl of the supernatant containing the bacterial DNA were transferred into sterile 1.5-ml tubes and frozen at -20°C until genetic analysis (procedure according to Millar et al. [32]).

##### 

**16S rDNA amplification** Independently of the identification with biochemical methods (STREPTOtest 24 Lachema, Pliva), the strains were identified based on their 16S ribosomal DNA sequence, aligned against the 16S ribosomal RNA sequences database (NCBI, National Center of Biotechnology Information) by BLAST.

For this purpose, the 16S rDNA fragment was amplified with use of universal primers (Forward: 5′-CGCTGGCGGCGTGCCTAATA-3′, and Reverse: 5′-TGCAAAGCAGGCGC-TCTCCC-3′). The PCR program, performed on the Eppendorf MasterCycler thermocycler, consisted of the following steps: Initial denaturation (95°C for 15 minutes) and 40 cycles of denaturation (95°C, 1 min), annealing (56°C, 15 min) and elongation (72°C, 2 min), followed by a final elongation step (72°C, 10 min). The reaction mixture at a final volume of 50 μl contained10 mM Tris-HCl (pH 8.3), 50 mM KCl, 1.5 mM MgCl_2_, 0.2 mM deoxynucleoside triphosphate, 0.2 μM of each primer, 1U Taq DNA polymerase (HotStarTaq®, Qiagen), and 100 ng bacterial DNA.

##### 

**Electrophoresis** The obtained PCR product was separated by electrophoresis (Biometra Horizon 20.25 with Consort EV243 power pack) on a 1% agarose gel with 4 μl ethidium bromide (10 mg/ml stock solution, Sigma-Aldrich) for 35 minutes (160 V). The products were visualised by a gel documentation system with UV transilluminator (UVP GelDoc IT310).

##### 

**Sequencing** After purification of the PCR product by use of a commercially available kit (PCR Purification Kit, Qiagen), the 16S rDNA fragment was sequenced: First, the reaction mix was prepared. The final volume of 15 μl contained 3 μl polymerase mix (BigDye® Terminator v3.1 Cycle Sequencing Kit, LifeTechnologies), 3 μl reaction buffer (BigDye® Terminator 5x Sequencing Buffer, LifeTechnologies), 3.2 μl of 1 μM F or R primer (the same as used for the PCR reaction), and 30 ng of the purified PCR product. All analyzed samples were sequenced in both directions – forward and reverse. Steps of the sequencing PCR: 96°C for 1 min, 25 cycles comprised of 96°C for 15 seconds, 54°C for 7 seconds, 60°C for 5 minutes. The obtained products were purified by ethanol precipitation: 2 μl sodium acetate/EDTA (1.5 M sodium acetate, pH > 8.0 and 250 mM EDTA) was added to each sample, followed by DNA precipitation with 80 μl of 95% ethanol (EtOH). The samples were mixed thoroughly and centrifuged for 15 minutes at 10,000 × g. After removal of the supernatant, the DNA pellet was rinsed with 70% EtOH and centrifuged briefly. The pellet was then air-dried and resuspended in 20 μl highly deionized formamide (Hi-Di™, LifeTechnologies) and immediately run on a sequencer (Genetic Analyzer 3500 Series (Hitachi) Applied Biosystems).

##### 

**Figure 1:**
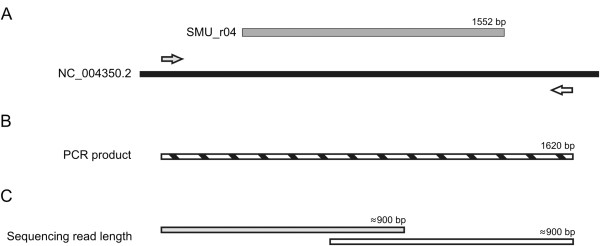
**16s rDNA sequencing strategy. A**. NC_004350.2 – *Streptococcus mutans* UA159 genome, SMU_r04 - 16S ribosomal RNA gene (length 1552 bp), grey arrow – forward primer (CGCTGGCGGCGTGCCTAATA), white arrow – reverse primer (TGCAAAGCAGGCGCTCTCCC). **B**. PCR product (length 1620 bp). **C**. Average sequencing read length for forward (grey stripe) and reverse (white stripe) primer.

**Sequence analysis** The primer pair was designed to give a product of about 1620 nucleotides in length in the case of *S. mutans* UA159 (NC_004350.2). A single read during capillary electrophoresis on the 3500 Series Genetic Analyzer unravels approx. 800-900 nucleotides. Therefore, the obtained 2 results (from the sense and the antisense primer) were assembled into one consensus sequence by bioinformatic tools (SeqScape v2.7 software, LifeTechnologies), exported into fasta format, and aligned against the 16S ribosomal RNA sequences database accessible through the NCBI (National Center for Biotechnology Information) website by Megablast (BLAST, Basic Local Alignment Search Tool) (schematic representation in Figure 1). The conformity of sequences obtained by BLAST served as basis for the identification of strains [33-35].

The statistical clusterization method was used in order to find similarities between the obtained profiles.

##### 

**Data accessibility** The full 16S rDNA sequences of the genetically analyzed strains are available at the National Center for Biotechnology Information GenBank database (http://www.ncbi.nlm.nih.gov/genbank/) under the following accession numbers: KM052277, KM052278, KM052279, KM052280, KM052281, KM052282, KM052283, KM052284, KM052285, KM052286, KM052287, KM052288, KM052289, KM052290, KM052291, KM052292, KM052293, KM052294, KM052295, KM052296, KM052297, KM052298, KM052299, KM052300, KM052301, KM052302, KM052303, KM052304, KM052305, KM052306, KM052307, KM052308, KM052309, KM052310, KM052311, KM052312, KM052313, KM052314, KM052315, KM052316, and KM052317. Sequences that are gapped in the region of the read merge (n = 2) are not included.

### Examination of prephenate dehydrogenase (PDH) activity

**Figure 2:**
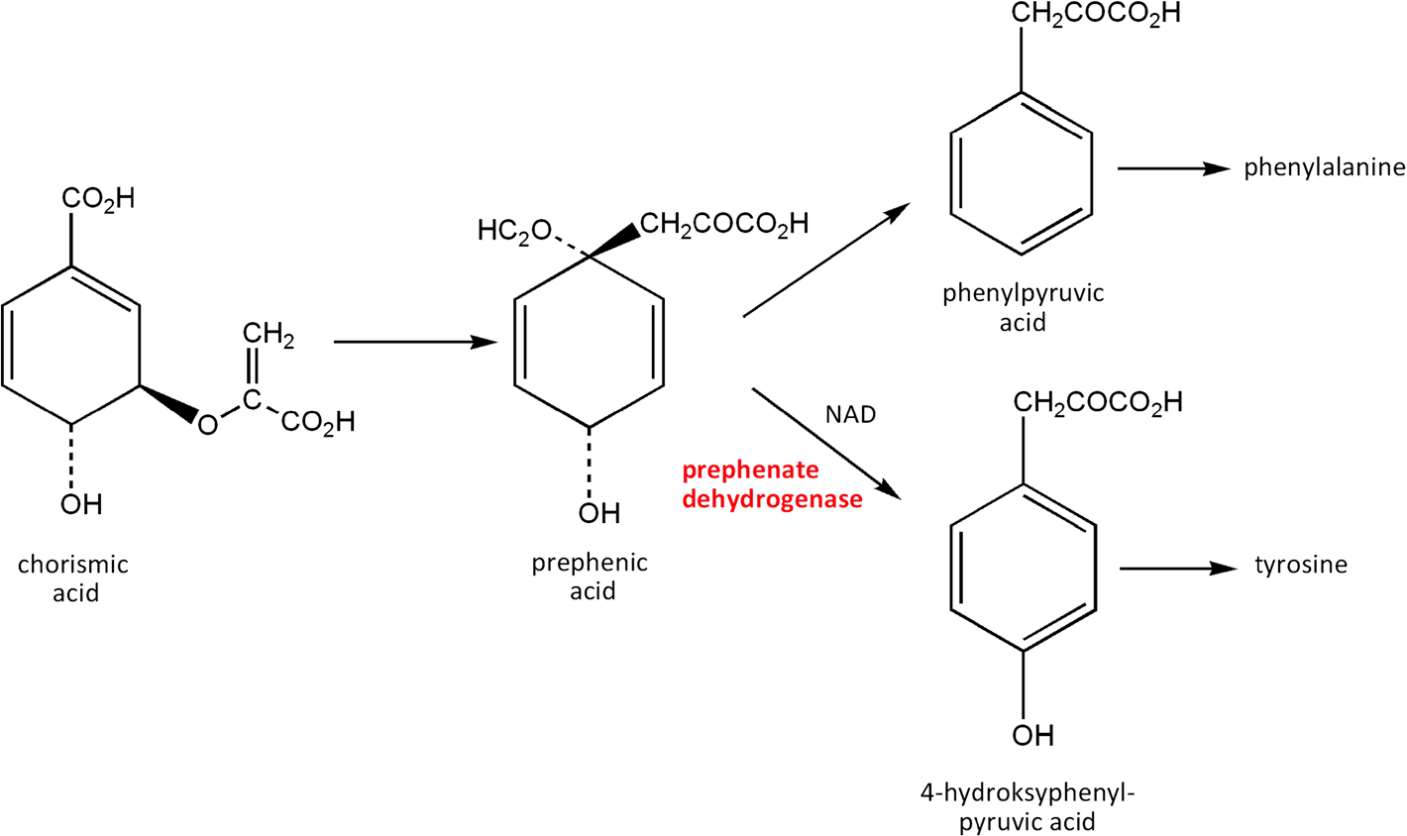
Role of the prephenate dehydrogenase (PDH) in the metabolism of aromatic amino acids.

Prephenate dehydrogenase (PDH) is an enzyme catalyzing the reaction of prephenate transition into 4-hydroxyphenylpyruvate, a compound from which tyrosine is directly synthesized (Figure 2). The subject of our research was PDH isolated from *S. mutans* strains. Enzymatic activity of PDH was determined fluometrically based on a method described by Champney and Jensen for *B. subitilis*[36], with modifications introduced by Ku et al. to determine the activity of the *S. mutans-*derived enzyme [9].

#### ***Bacteria preparation***

Pure bacterial colonies (after 48 hours culturing on BA medium in conditions of 10% CO_2_ at a temperature of 36°C) were suspended in 12 ml of sterile PBS solution, and centrifuged 4°C, for 15 min at 3000 × g. The cell pellet was suspended in 3 ml of phosphate buffer (0.05 M, pH = 7.8) containing KCl (0.1 M) and lysozyme (100 μg/ml). The bacterial mixture after addition of lysozyme was incubated at a temperature of 37°C for 15 min. After incubation, DNase at a final concentration of 10 μg/ml was added to the mixture. Cell remnants were removed by centrifugation at 12000 × g at 4°C, for 20 minutes. 3 ml of the supernatant was spread on a column containing Sephadex G-25 (1.5 × 7.4 cm). The protein eluate (2.5 ml) was collected for PDH examinations [36,37].

#### ***PDH activity determination***

PDH activity was examined fluorimetrically at an excitation wavelength of 340 nm and emission at 460 nm for 5 minutes (F-2000 fluorimeter, Hitachi). The result was expressed as a decrease in fluorescence per 1 minute. 1 ml of the reaction mixture (0.79 mg nicotinamide adenine dinucleotide (NAD) (1.2 μM); 2.42 mg Tris-HCl (20 μM, pH = 6), 4.473 mg KCl (60 μM); 4.083 mg K_2_PO_4_ (30 μM,) and 50 μl prephenate dehydrogenase) was incubated for 3 minutes at 37°C in a heating block. The reaction was initiated by addition of 0.361 mg of prephenate (1 μM) (prephenic acid barium salt, No.: P2384, Sigma-Aldrich, Poland). The reduced nicotinamide adenine dinucleotide - NADH formed during the reaction was measured. The reaction was terminated by addition of 1 ml NaOH (1.5 M) to the reaction mixture [9,36,38].

### Statistical analysis

Analysis of variance using the Brown–Forsythe test was applied for comparison of the activity of prephenate dehydrogenase (PDH) isolated from clinical strains of *S. mutans* in patients with caries (the examined group) and without disease symptoms (the control group). Conformity with a normal distribution (p > 0.05; Shapiro–Wilk test), as well as non-uniformity of variance in the groups (p < 0.05) were the basis for the application of a parametric test with separate variance assessment for independent samples (examined and control groups) - the Cochran and Cox test.

The values of the medians of PDH activity in the established biotypes (I-III) were compared. The non-parametric Kruskal-Wallis test for independent samples was applied. Normality of distributions in the examined groups (proposed biotypes) was verified using the Shapiro-Wilk test. Analysis of variance using the Levene test was applied for comparison of the activity of PDH between specific biotypes.

The statistical clusterization method was used in the case of biotyping. The results of the division into clusters were presented in the form of diagrams - dendrograms. The data were elaborated statistically using R language version 2.10.1 and an environment for statistical calculations in the Linux operating system, and also in Statistica v.10 PL software (StatSoft, Poland). Statistically significant differences were noted in the case of all analyzed groups (p < 0.001).

### Ethical approval

Consents were obtained from all patients, and the study procedure was approved by the Bioethical Committee of the Jagiellonian University in Krakow (No. KBET/171/B/2011).

## Results

### Characteristics of isolated strains

**Figure 3:**
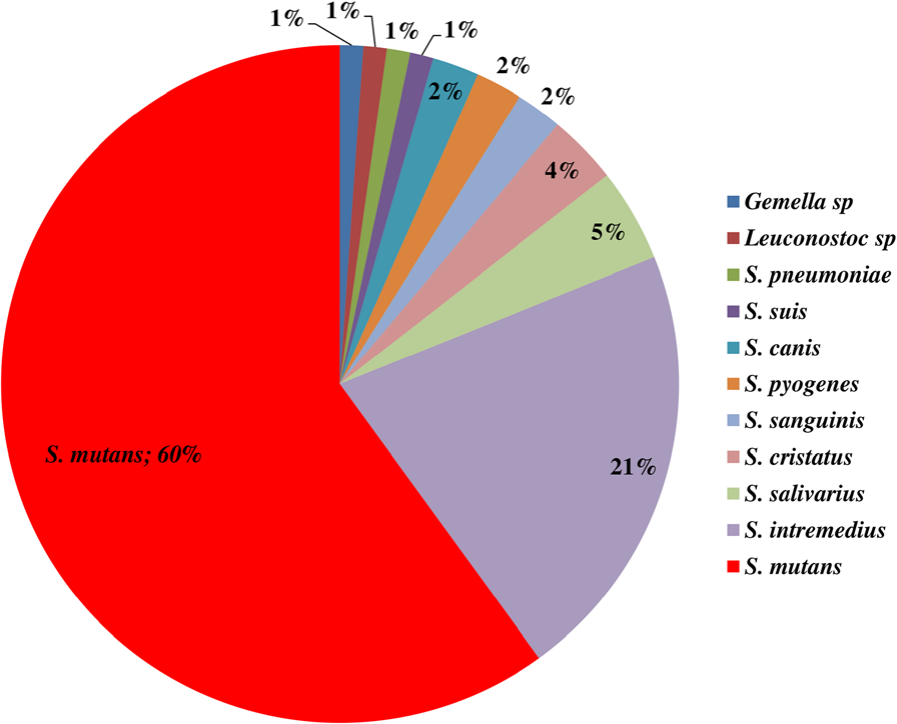
**Percentage of Streptococcus species in the material using a selective medium HLR-S.** The results of phenotypic analysis using the biochemical test.

In total, 90 strains belonging to 10 Streptococcus species were isolated from patients and controls, as well as 1 strain belonging to the *Gemella* species. The *S. mutans* was the most abundant (53 strains), followed by *S. intermedius* (20 strains). The percentage contribution of particular species is presented in Figure 3.

The strains originated from carious foci of childrens’ decidous teeth (n = 61) with diagnosed early childhood caries (ECC) - the examined group, and from dental plaque of children (n = 29) without carious changes in decidous teeth - the control group.

**Figure 4:**
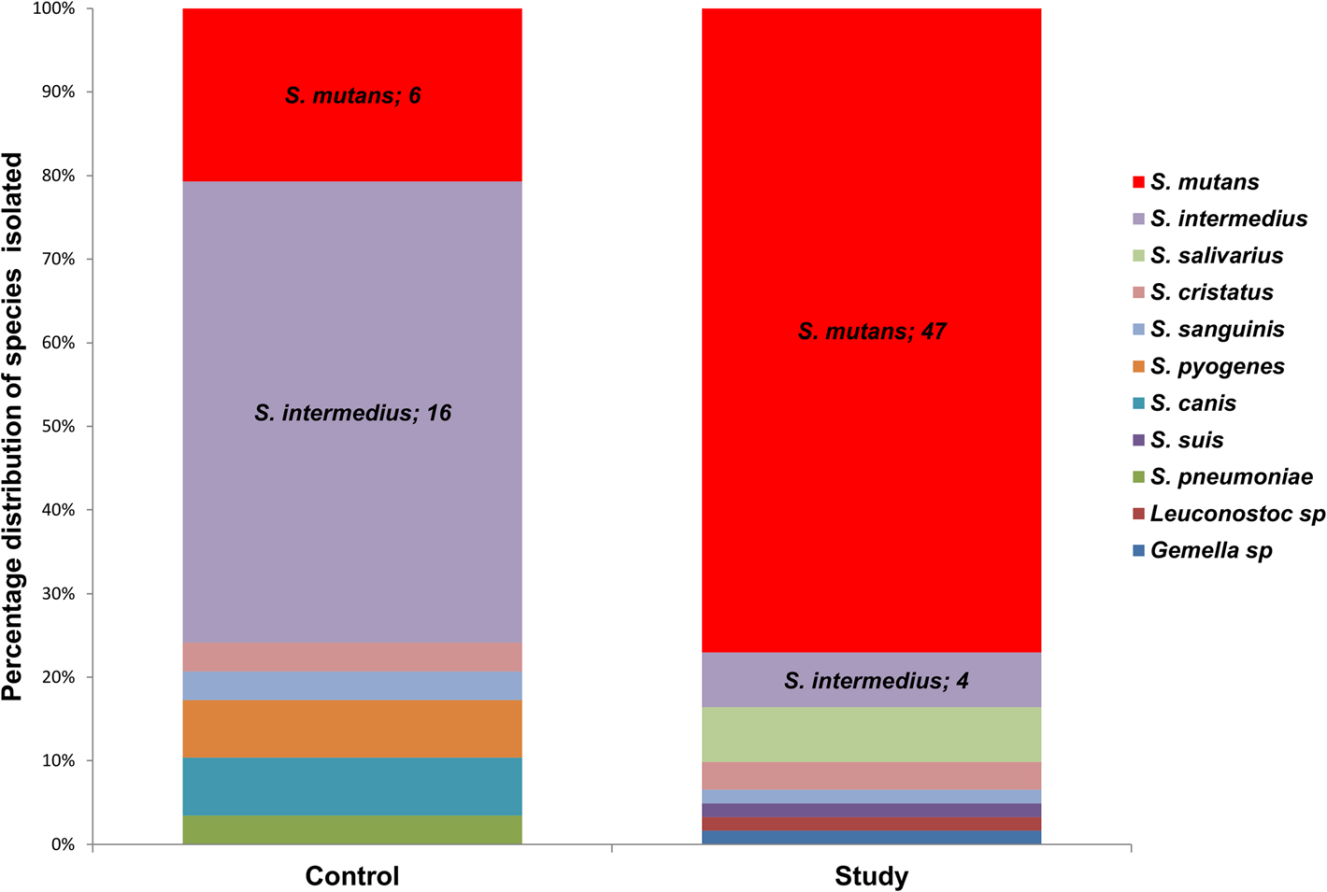
Range and relative share of Streptococcus species in groups - study and control.

The contribution of the Streptococcus species in both the examined and control populations was different; the frequency of *S. mutans* isolation in the particular groups is presented in Figure 4.

**Figure 5:**
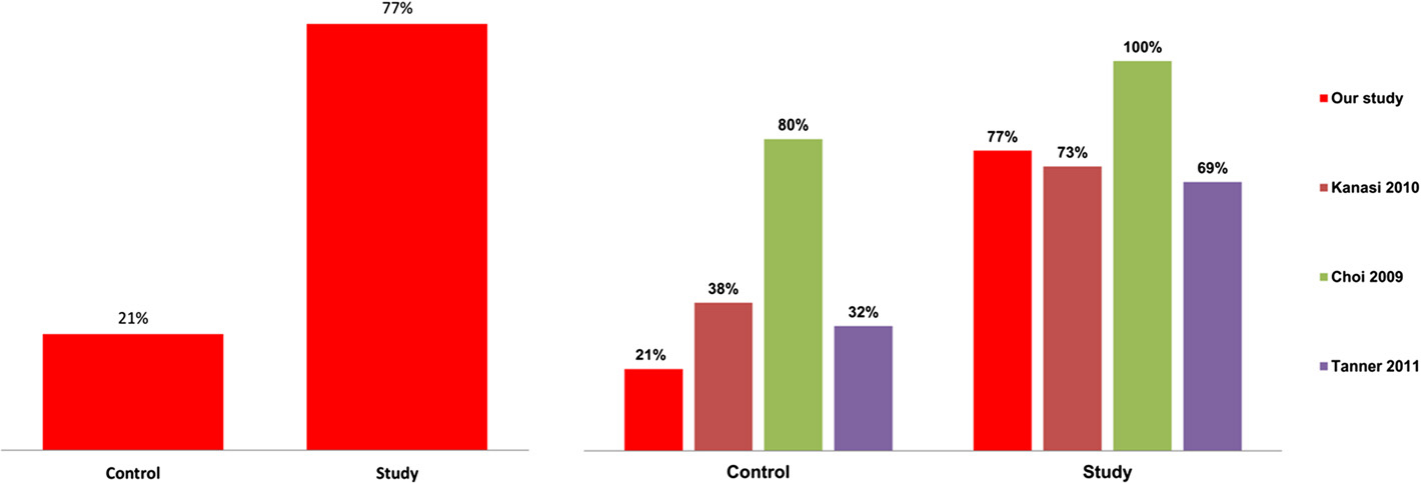
**The incidence of *S. mutans *species divided into groups.** On the left, the results obtained in our study; on the right, a comparison of our results with those of other authors.

The predominance of *S. mutans* in caries in children was confirmed in the present study where it was isolated from 77% of carious foci. Selective HLR-S medium (a modified Ritz medium) was applied in order to isolate the species from clinical material. Diagnostic sensitivity for the above medium was 77%, while diagnostic specificity was 76%. The obtained frequency of *S. mutans* isolation from children with caries (77%) is at a similar level to that identified in the study by Kanasi et al. – 73% [4] (Figure 5).

The percentage of *S. mutans* strains observed in the examined group with caries was over three-fold higher than in the control group (Figure 5), which confirms the relationship between the presence of *S. mutans* species and early childhood caries. Comparing the results obtained with current literature reports (Figure 5), a similarity in the frequency of *S. mutans* occurrence in children with caries may be noticed. In material derived from children without caries (n = 29) *S. mutans* was isolated from 6 samples, i.e. 21%. This value is lower than the results obtained in similar research worldwide. This fact may be explained by the application of more sensitive methods in the other studies. Choi et al., as well as Kanasi et al., used genetic methods in their studies for direct identification of the bacterial flora of children’s oral cavity. Tanner et al., based on “classic” microbiology methods - microorganism culture, obtained results close to those obtained in the present study (32%). The higher frequency of *S. mutans* isolation obtained in the study by Choi et al. may be connected to environmental predispositions or different feeding habits in the examined groups.

### 16S rDNA genotyping

Out of 90 isolated strains, which were typed biochemically with STREPTOtest24, the 16S rDNA sequence was determined in strains with a discrimination rate of less than 95%. Thirteen strains were not genotyped because we failed to isolate DNA. In total, the 16S rDNA sequence was analyzed for 42 strains with a discrimination rate of less than 95% and for 2 randomly chosen strains with a discrimination rate of over 95%, in order to confirm the arbitrally accepted threshold.

The genetic typing confirmed the biochemical test in 75% of strains (32 of 42 genetically tested strains).

For both randomly chosen strains the results obtained by biochemical and genetical methods were unanimous. In the first case, *S. intermedius* was revealed by the STREPTOtest24 with a determination rate of 96.15%, and by genotyping based on a 99% identity with the 16S rDNA sequence from *S. intermedius*. The second case was designated as *S. intermedius* at a discrimination rate of 97.57% by STREPTOtest24 and 100% by sequencing.

The genetic analysis confirmed 2 cases among 7 (29%), for which the biochemical identification showed low discrimination rates (71.14% for *S. salivarius*, 55.4% for *S. canis*, 52.67% *S.pyogenes*, 52.27% *S. intermedius*): in one case, where *S. salivarius*/*S. mutans*/*S. sanguinis* were typed biochemically with the probability of 71.14, 10.96 and 10.14%, respectively, the genetic analysis confirmed the presence of *S. salivarius*. In two cases with a low discrimination rate of 55.40/33.69/10.14% for *S. canis*/*S. sanguini*/*S. intermedius* respectively, the analysis of DNA revealed the presence of *S. intermedius*. Two other cases, phenotypically designated as *S. pyogenes/S. canis/S. sanguinis* with the probability of 52.67/11.73/10.70% respectively in the STREPTOtest24, were genetically equal to *S. intermedius*. One strain identified biochemically as *S. intermedius/S. pneumoniae*/*S. sanguinis* (showing similarly low discrimination rates of 52.27/16.24/10.08%) genetically turned out to belong to the *Lactobacillus* species (*L. rhamnosus*).

The biochemical result for another tested sample (encoded 20 K) were as follows: *S. canis*/*S. sanguini*/*S. intermedius* (55.40/33.69/10.14%), which could not be confirmed genetically. The length of the amplified 16S rDNA read on the Genetic Analyzer was 1409 base pairs. The sequence of the obtained 16S rDNA for the above-mentioned sample (20 K) was shown by BLAST to be 99% identical to the 16S rDNA of *S. intermedius*. Therefore, the strain was eventually identified as *S. intermedius*.

### Biotyping

Due to the small amount of isolates belonging to *S. pneumoniae*, *S. canis*, *S. cristatus*, *S. suis*, *S. saniguinis*, *Gemella sp*., *Leuconostoc sp*., *S. pyogenes*, *S. salivarius S. intermedius*, biotypes were determined only for *S. mutans*. Two approaches to the determination of biotyping criteria, based on the profiles of enzymatic activity of the STREPTOtest 24 test, were applied.

**Figure 6:**
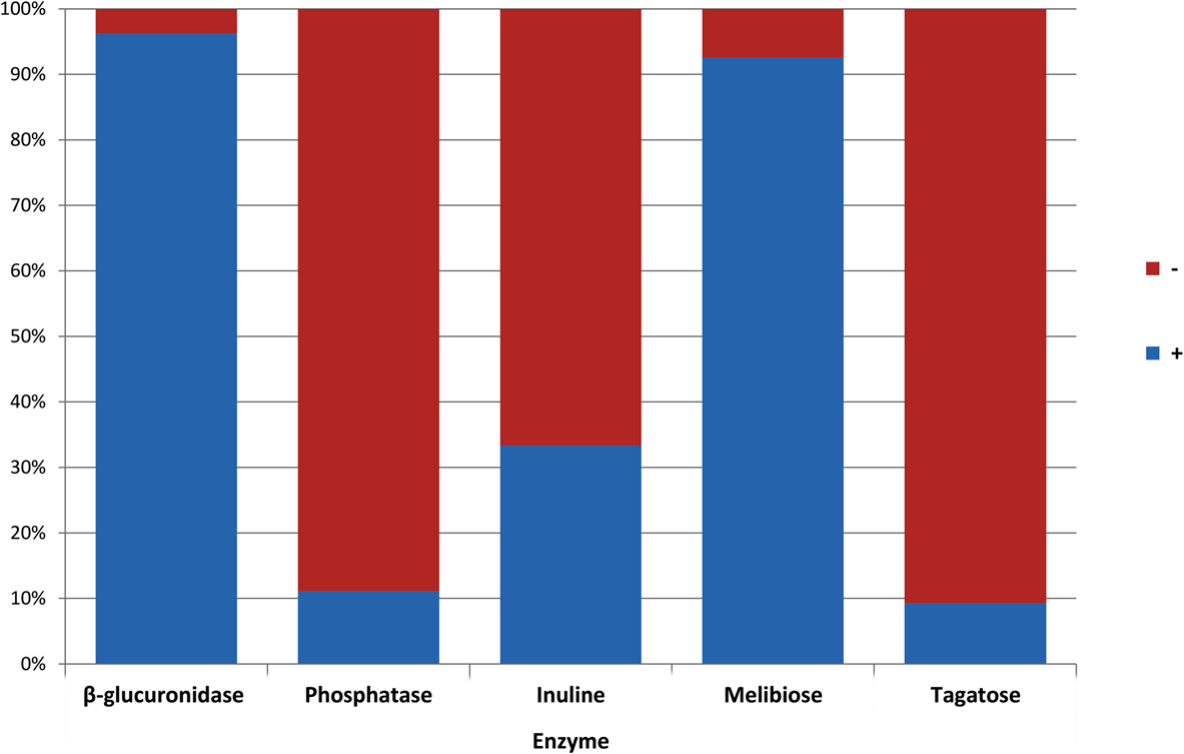
Distribution of enzymes selected for biotyping (graphs marked in blue).

The first approach involved the selection of suitable enzymes based on the analysis of their activity in the population of the examined strains. Ten enzymes present in all the strains (reactions assigned in Table 3: LAP, bGA, Aga, ESL, MAN, SOR, LAC, MLT, RAF, TRE) were not suitable for differentiation and were rejected *per se*. These were tested on the basis of their growth in the presence of 6.5% NaCl which was negative in all the strains, and 7 enzymes which were not noted in the strains (bMN, GLR, RIB, PUL, ARG, AMG, SOE). Another two enzymes (β-glucuronidase, observed in 96.3% of the strains, and N-acetylglucosaminidase, present in 98.15% of the strains exhibiting this enzyme activity) were observed only in one case and were also rejected since their application in biotyping would cause the distribution of strains in particular biotypes to be non-uniform, i.e. an overly large number of biotypes would be present for which only one strain would be classified. Another 4 enzymes seemed to be suitable for biotyping. Their occurrence among the strains is within the quartile deviation - between 5.95 -94.05%. These are: phosphatase 11.11%, inulin– 33.33%, melibiose – 92.59%, tegatose – 9.26% (Figure 6).

The acceptance of these enzymes as biotyping criteria allowed for the definition of 7 enzymatic profiles, which are conventionally designated using sequential alphabetical lettering from A to G, as presented in Table 4. The most abundant was profile A characterized by a lack of phosphatase, inulin and tegatose activity.

**Table 4:** ***S. mutans *** **biotypes obtained based on the activity of four enzymes**

**Enzyme profile**	**PHS**	**INU**	**MLB**	**TGT**	**Number of strains**	**Participation %**
A	-	-	+	-	31	58%
B	-	+	+	-	13	24%
C	+	+	-	-	3	6%
D	-	-	+	+	3	6%
E	+	-	+	-	1	2%
F	+	-	+	+	1	2%
G	-	+	+	+	1	2%

The distribution of the profiles thus obtained was non-uniform; therefore, strains differing only in terms of one reaction were grouped in one biotype. The biotypes presented in the table below were obtained (in Table 5).

**Table 5:** **Differentiation of *S. mutans *** **biotypes based on differences in the activity of phosphatase, inulin, tegatose and melibiose**

**Biotype**	**Enzyme profile**	**PHS**	**INU**	**MLB**	**TGT**	**Number of strains**	**Participation %**
I	A + D	-	-	+	-/+	34	64%
II	B + G	-	+	+	-/+	14	26%
III	C	+	+	-	-	3	6%
IV	E + F	+	-	+	-/+	2	4%

Biotype I was observed in 64% of cases, was characterized by a lack of phosphatase (PHS) activity and a lack of inulin (INU) decomposition, however it demonstrated the presence of a melibiose (MLB) decomposing enzyme, and tegatose (TGT) decomposition was a variable feature. Biotype II observed in 26% of cases was characterized by decomposition of inulin (INU) and tegatose (TGT) with a lack of phosphatase (PHS) activity; tegatose (TGT) distribution was not uniform within the biotype. Biotype III (6%) exhibited the activity of phosphatase (PHS) and enzyme decomposing inulin (INU), reactions of melibiose (MLB) and tegatose (TGT) fermentation were negative. The least abundant was biotype IV, characteristic for 4% of the examined strains, which demonstrated phosphatase (PHS) activity, decomposed melibiose (MLB) but not inulin (INU), while tegatose (TGT) decomposition was variable.

**Figure 7:**
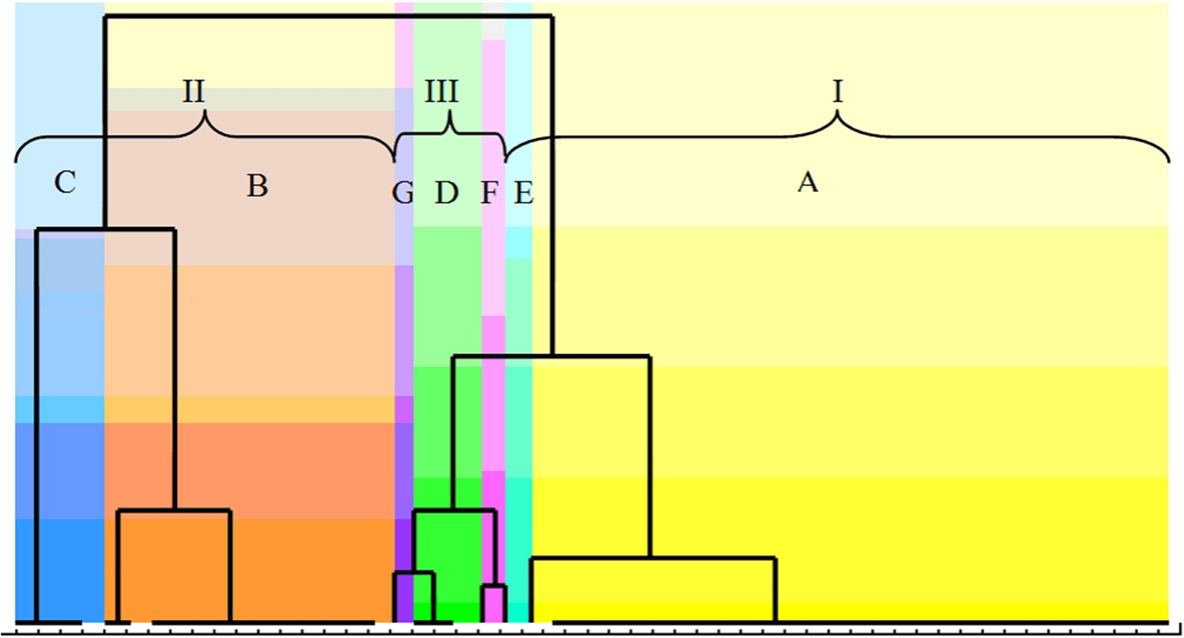
**Dendrogram with marked *S. mutans *** **biotypes.**

The second method of biotype determination was the application of non-supervised statistical data analysis (without *a priori*-available knowledge). In this method the data were divided into groups (clusters) so that each of the groups was as uniform as possible (strains within the groups were similar), and concurrently the clusters were diversified (strains from various groups had the least possible number of common features).The results of division into clusters are presented in the form of diagrams - dendrograms. In the case of the most adjusted dendrogram it appeared that the strains may be differentiated based on the activity of inulin, melibiose and tegatose. This allowed 3 biotypes to be separated - the branches described with Roman figures (I-III) on the diagram (Figure 7). The Figure also shows the distribution of enzymatic profiles obtained using the arbitrary method.

### Enzymatic activity of prephenate dehydrogenase (PDH; *EC 1.3.1.12*)

**Figure 8:**
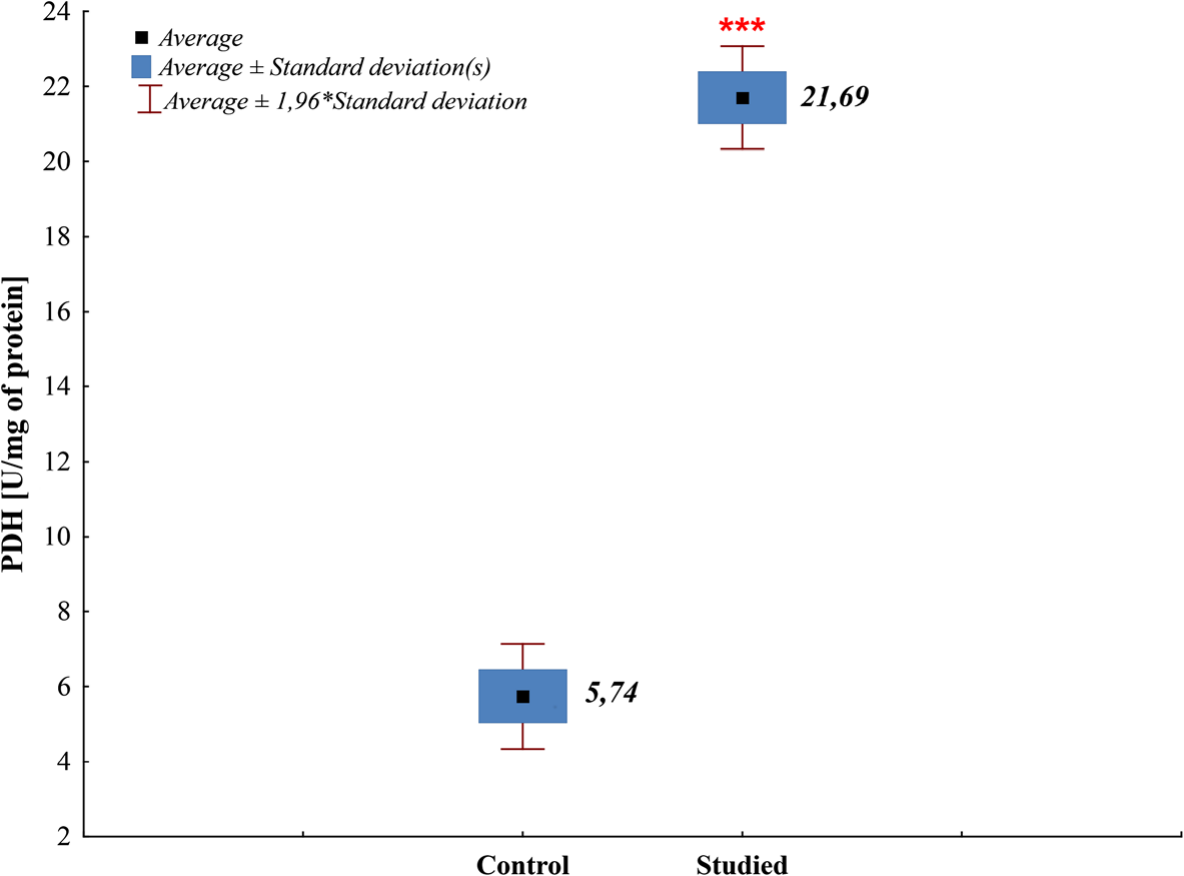
**Graphical interpretation of the Cochran-Cox test, p <0.001.** Comparison of average prephenate dehydrogenase enzymatic activities, PDH in the control and study group; *** - p <0.001.

Enzymatic activity of prephenate dehydrogenase (PDH) was determined for clinical *S. mutans* strains (n = 53). The existence of relationships was examined between PDH activity in bacteria and caries presence in the patient the strain was isolated from. The parametric test with separate variance assessment for independent samples (examined and control groups) - the Cochran and Cox test, was applied to compare the activity of prephenate dehydrogenase PDH between the examined and control groups. Mean enzymatic activities of prephenate dehydrogenase PDH in the control and examined groups differed statistically (Cochran-Cox test, p < 0.001) (Figure 8).

**Figure 9:**
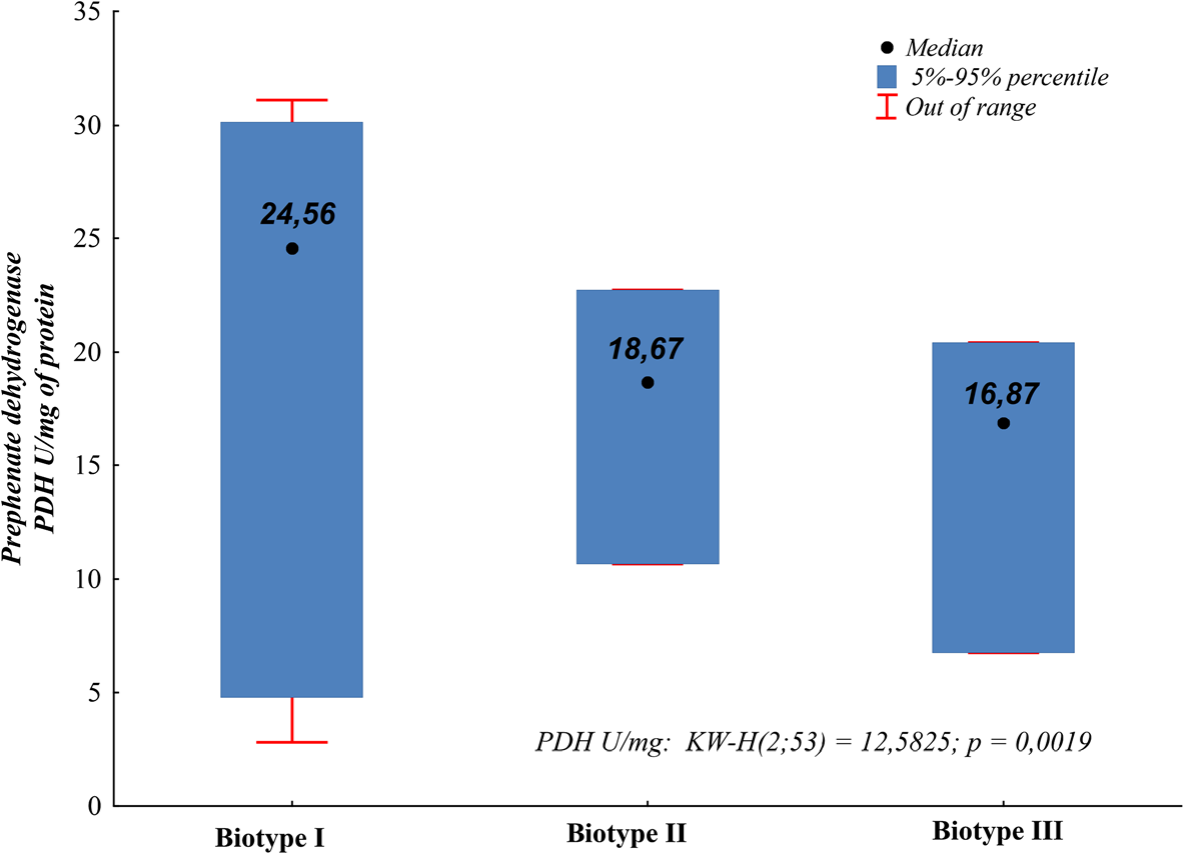
**Graphical interpretation of the Kruskal-Wallis test, p = 0.0019.** Comparison of median values of prephenate dehydrogenase (PDH) in biotypes (I-III).

The enzymatic activity of PDH was compared in the context of *S. mutans* biotypes I-III proposed in the study (clusterization method). The non-parametric Kruskal-Wallis test for independent samples was applied in order to compare the activity of prephenate dehydrogenase PDH between particular biotypes (biotype I-III). Attention was paid to the presence of statistically significant differences (Kruskal-Wallis test, p = 0.0019) (Figure 9).

## Discussion

### Occurrence and characteristics of the isolated strains

Since the main source of *S. mutans* infections is the oral cavity, it seems to be significant to determine what percentage of *S. mutans* strains inhabiting the human oral cavity and its direct vicinity possess the features determining their ability to cause caries.

#### ***Occurrence of Streptococcus mutans strains***

Cases of early childhood caries of deciduous teeth described in casuistic studies are most often caused by the *S. mutans* species [4]. The frequency of species occurrence is variable and ranges from 69% [5] to 100% [39]. In the event of an absence of *S. mutans*, the species isolated the most often from children with caries are: *S. gordonii*, *S. cristatus* and *S. intermedius *[5].

In the whole examined population, the *S. mutans* species were isolated from 60% of study participants. The obtained result is consistent with the results obtained by Brauncajs et al [40].

Apart from *S. mutans* strains, other species belonging to the Streptococcus genus were also identified in the present study, mainly *S. intermedius*, *S. salivarius*, *S. cristatus* (Figure 3). The conducted study confirms that the presence of pathogenic bacteria itself is not sufficient for caries development - *S. mutans* strains were cultured from 21% of the children classified by a dentist as “healthy”.

### Biotyping

The biotyping of *S. mutans* species is an interesting issue which focuses attention of researchers all over the world [22,41,42]. Apart from the availability of genetic methods allowing better differentiation of the species, e.g. for epidemiological purposes [26], typing on the biochemical level, which due to low cost is a competitive method, is still an open issue. There are however no biotypes, and thus no enzymes determined in the literature, on the basis of which biotyping for *S. mutans* would be possible. We noted that the most suitable enzymes for strain differentiation are: phosphatase (PHS), inulin (INU), melibiose (MLB) and tegatose (TGT).

Comparing the reaction results for the mentioned compounds obtained in our study with results obtained by Yoo et al. [22] it appeared that 50 out of 54 strains demonstrated the same profile. The scheme proposed by us and based on the metabolism of inulin, melibiose, tegatose and phosphatase activity is more adequate for establishing *S. mutans* biotypes in the Polish population. Phosphatase present in the strains isolated by us was characterized by variable activity, therefore that feature was used for biotyping.

The method seems to be promising in the context of recognition of mechanisms of vertical and horizontal bacteria transmission. It would be worth verification in further studies to ascertain whether the proposed biotypes are characteristic for related persons (mother - child, siblings).

The frequencies of occurrence of particular enzymes tested by Facklam et al. [41] were comparable with those obtained in our study, i.e. above 95%. In the case of melibiose decomposition, 93% of examined strains demonstrated the activity of a suitable enzyme, while Facklam observed that feature in nearly half of the strains isolated from the plaque (43%) and 88% of strains isolated from blood.

Melibiose decomposition is used in *S. mutans* differentiation [22], and the selection of that feature seems to be suitable for typing based on biochemical profiles. It should, however, be noted that strains not fermenting melibiose are a genetically non-uniform group.

Another way of establishing biotypes, i.e. clustering of “similar” bacterial strains, is the application of the statistical method of clusters analysis - clusterization. The clusterization method allows the activity of all enzymes of the STREPTOtest 24 test to be taken into account in defining biotypes. It appeared that the best differentiation of biotypes may be obtained based on occurrence of the activity of three enzymes decomposing INU, MLB and TGT. The obtained biotypes appeared to be useful in the differentiation of the strains in terms of PDH activity. Attention was paid to the existence of a relationship between PDH activity and affiliation to a specified biotype (Kruskal-Wallis test, p < 0.05). All the proposed biotypes were characterized by sufficiently frequent occurrence, the least abundant biotype III included almost 10% of the examined strains (9.25%).

### Enzymatic activity of prephenate dehydrogenase (PDH)

The study conducted is the first one, which examined the enzymatic activity of the PDH enzyme isolated from *S. mutans* clinical strains. Attention was paid to the existence of intra-species differences in the degree of enzyme activity (Min. - Max: 2.78-31.09 U/mg), which may be explained by the dissimilarity of the patient oral cavity environments the strains were isolated from (different microbiological flora, different chemical composition of saliva, etc.).

Our study indicates the existence of a relationship between enzyme activity in microorganisms and caries occurrence. Mean enzyme activity among the strains derived from carious foci was 21.69 U/mg, while the strains derived from healthy oral cavities demonstrated a 4-fold lower enzyme activity (5.74 U/mg) (Figure 8). Among the strains isolated from healthy children, PDH activity did not exceed 10 U/mg (maximum 7.58 U/mg). In the case of strains isolated from carious foci all demonstrated PDH activity above 10 U/mg (Figure 8). Possibly, there is a critical value (threshold level) of enzyme activity, beyond which an increase in microorganism virulence is noted. Further studies should be performed in order to establish whether the described differences in PDH activity are of a genetic or environmental background. Low enzyme activity is connected to *S. mutans* strains inhabiting healthy oral cavities. It is interesting, whether prevention in the form of enzyme inhibitors in the case of the control group would slow down, and as a consequence inhibit disease development in the future. Based on the study, it should be supposed that synthesis of tyrosine, one of the aromatic amino acids connected to prephenate dehydrogenase metabolism, may be one of the factors playing a role in *S. mutans* pathogenicity, and thus disease development in children.

PDH activity should be considered in a wider context, as a component of the whole bacterial metabolism also including other biochemical pathways. This is confirmed in the present study by the demonstrated correlation between the degree of PDH activity and affiliation of the strain to one of the proposed *S. mutans* biotypes (I).

Prephenate dehydrogenase (PDH) is absent in humans and therefore, according to Ku et al., could be a target for therapeutic specific inhibitors [9].

Various attempts at caries prevention are being widely undertaken all over the world: there is a search for anti-caries substances in food products such as black tea [43], as well as compounds against acid-forming bacteria acting in low pH are being synthesized [23], and the application of mutated *S. rattatus* strains as probiotics in caries prevention has also been proposed [23,44]. The present study may be a starting point in the creation of an alternative way of limitation, or even elimination, of the pathogenicity of *S. mutans* strains as a result of minimization of prephenate dehydrogenase (PDH) activity. The structure of the enzyme is known [45]. The synthesis of specific PDH inhibitors should be considered, and further studies aimed at verification as to whether this type of activities may prevent caries formation should be performed.

## Conclusions

*S. mutans* species is predominant among bacteria of the Streptococcus genus in early childhood caries (ECC) in carious foci. *S. mutans* strains may be differentiated into biotypes based on the profiles of enzymatic activity of the STREPTOtest 24 test. The prephenate dehydrogenase activity of most *S. mutans* strains from children with caries was higher than prephenate dehydrogenase activity demonstrated by compared *S. mutans* strains isolated from the children without disease symptoms. This may suggest a key role of that feature as a determinant of bacterial pathogenicity. Attention should be paid to the usefulness of biotyping in determination of selected pathogenicity determinants - prephenate dehydrogenase PDH activity in *S. mutans.*

Our results prompt further studies to be undertaken to confirm or exclude the pathogenicity of selected Streptococcus strains in an animal research model, and also cast new light on the formation of potential compounds blocking the metabolism of aromatic amino acids (e.g. PDH inhibition) which are connected to the life cycle of *Streptococcus mutans*.

Current studies on PDH isolated from *S. mutans* indicate that this enzyme has a homodimeric structure and each dimer consists of two domains: an N-terminal domain binding NAD, and a C-terminal domain binding prephenate [45]. Literature data indicate that enzyme activity is the highest at pH 6.8 or lower. The concentration of NaCl does not have any effect on PDH activity. No compounds were found in literature that could decelerate or modify the activity of prephenate dehydrogenase. In our studies, enzymatic activity in the range pH 5.0 – 6.0 was demonstrated. An intense decrease in PDH activity was observed at pH > 6.0. Some authors have assumed that PDH activity can be inhibited *via* the feedback inhibition of tyrosine [10]. However, there is insufficient data to support such a hypothesis.

The present observations are the first which demonstrate the relationship between one of the potential factors determining the pathogenicity of the *S. mutans* species - the activity of prephenate dehydrogenase (the key enzyme of aromatic amino acid metabolism) and biotyping of the *S. mutans* species based on phenotypic features (biochemical profiles). The results obtained this way may allow for better differentiation of the *S. mutans* species and thus may contribute to better recognition of the mechanisms of the transmission of pathogenic bacteria inhabiting the human oral cavity and facilitate treatment.

## Competing interests

All authors declare that they have no competing interests.

## Authors’ contributions

WK is the person behind the idea of the study, and participated in its design, prepared and analyzed samples, performed all non-genetic experiments during the study. WK also performed the statistical analysis and data interpretation, and was responsible for the manuscript preparation. KKP participated in study design, prepared the samples and performed the phenotyping experiment with the data analysis. JP, AS participated in study design, prepared the samples and performed the genotyping experiment with the data analysis. AS is additionally responsible for corrections leading to the final version of the manuscript. PK participated in study design. AJ participated in study design. DK participated in study design and collected clinical material. All the authors read and approved the submitted version of the manuscript to the journal.
